# Risk factors for viral hepatitis A infection in Gampaha District, Sri Lanka: an unmatched case control study

**DOI:** 10.1186/s12889-020-08490-2

**Published:** 2020-03-18

**Authors:** Nalin Ariyarathna, Chrishantha Abeysena

**Affiliations:** 1 Senior Registrar in Community Medicine, Ministry of Health, Sri Lanka; 2 Senior Professor in Community Medicine, Department of Public Health, Faculty of Medicine, University of Kelaniya, Kelaniya, Sri Lanka

**Keywords:** Water, Hepatitis, Hygiene, Risk, Sanitation

## Abstract

**Background:**

Hepatitis A virus (HAV) is one of the commonest food and water borne infectious diseases. The objective of the study was to determine the risk factors of HAV infection in the Gampha District in Sri Lanka.

**Methods:**

This was an unmatched case control study conducted between January 2015 and November 2016 comprising of 504 participants with a case control ratio of 1:1. The study population included individuals of age 1 year and above who were permanent residents of the district. Cases included participants admitted to four secondary care state hospitals with an acute HAV diagnosed by detecting serum anti-HAV IgM antibodies. Controls were randomly selected individuals from the community with serum negative for Anti-HAV IgM and IgG. An interviewer administered questionnaire was used for the data collection and multiple logistic regression was applied to determine the independent risk factors. The results are expressed as adjusted odds ratios (AOR) and 95% confidence intervals (CI).

**Results:**

Risk factors for HAV infection were poor knowledge regarding hepatitis (AOR;3.98, 95% CI = 1.97–8.05), unhygienic sanitary practices (OR = 2.73; 95% CI = 1.42–5.23), unhygienic practices related to drinking water (OR = 2.67; 95% CI = 1.37–5.21), residing in urban areas (OR = 5.94; 95% CI = 2.98–11.86) and lower family income (OR = 2.83; 95% CI = 1.30–6.13).

**Conclusions:**

The independent modifiable risk factors for HAV infection were poor knowledge regarding hepatitis, unhygienic sanitary practices, and unhygienic practices related to drinking water. Community awareness must be raised on hygienic practices and safe water drinking practices. Inequities of social determinates of health must be addressed.

## Background

Acute viral hepatitis is a systemic illness that predominantly affects liver causing clinical symptoms that include jaundice, dark urine and tender hepatomegaly with fever. Hepatitis A, B, C, D, and E are responsible for almost all the cases of viral hepatitis. The sero-prevalence of Hepatitis A virus (HAV) was 80.7% in Sri Lanka (14), 63.8% in Korea (12) and 41.1% in Australia (13). Generally, HAV infections are self-limiting without symptoms or with non-specific symptoms, and a low mortality. More severe cases of HVA are admitted for inward care and given supportive treatment combined with close monitoring. Nevertheless, for those who experience symptomatic illness, treatment costs, loss of productivity and human suffering can be significant [1–3].

According to a study, young children are mostly asymptomatic leading to a higher spread of the disease [4] and several studies reported that the risk of hepatitis A increases with age [5–7]. Other associated factors were living on a low family income [8, 9] and lower educational level [9, 10]. The transmission of Hepatitis A occurs most commonly through the faeco-oral route and consequently, consumption of contaminated food and water plays an important role in the spreading of HAV. Consumption of contaminated food was identified as the commonest cause for both sporadic cases and outbreaks of HAV [4, 11]. One study carried out in 2004 identified not having access to tap water as a risk factor for hepatitis A infection [12]. A number of studies showed that poor sanitation was a risk factor for HAV infection [12, 13]. In addition, it can be expected that the peoples’ knowledge regarding HAV and the spread of the disease can be thought to be associated with the prevalence of HAV infection.

Despite the fact that the risk factors for HAV are well recognized globally, to the best of the author’s knowledge, no studies have been carried out to identify risk factors for HAV in Sri Lanka. The district of Gampaha is one of the most populous districts in Sri Lanka and it was felt important to assess the risk factors for HAV in the area. Determining the risk factor profile of viral hepatitis may well avert the occurrence and spread of disease by the introduction of rational preventive strategies. Since studies to identify HAV risk factors were not conducted in Sri Lanka prior to this, this study would explore the much needed data for HAV disease prevention. Hence, studying the risk factors comprehensively helps to formulate well focused cost effective preventive strategies to prevent HAV infections. Results of the proposed study would provide knowledge for proper planning for the prevention of the disease by risk factor identification. The objective of the study was to determine the risk factors for viral hepatitis A of a district in Sri Lanka.

## Methods

An unmatched case control study was done recruiting hospital based cases and community based controls from the district of Gampaha between January 2015 and November 2016. The study population included individuals of age 1 year and above who were permanent residents of the District. A case was defined as a patient who was serologically confirmed as having HAV infection by detecting serum anti-HAV IgM antibodies by ELISA testing. A control was an individual who was serum negative for Anti-HAV IgM and IgG. Patients with severe psychiatric illnesses were excluded since the reliability of data was a concern. The cases were selected from four secondary care state hospitals and the controls from the community. The cases were selected consecutively during the study period. The Controls were individuals who were permanent residents in the district of Gampaha at the time of data collection. The controls were selected using a multi-stage stratified cluster sampling technique. The selection of the controls has been described elsewhere [14].

The sample size was calculated considering the exposure among the controls as 0.55, the odds ratio as 1.75 [15], desired probability of type I error as 0.05 and the power as 0.80. Therefore, the minimum of 221 samples of cases and controls were needed. Considering the non-response rate of 15%, the required sample size was 260 for a group.

Data were collected through an interviewer-administered questionnaire (IAQ). It covered socio-demographic characteristics, knowledge of viral hepatitis, past clinical information of viral hepatitis, and risk factors of HAV. The information on history of viral hepatitis and clinical manifestations were confirmed only if documentary evidence were available.

The probable risk factors for viral HAV were searched on literature and they were found to be poor knowledge regarding viral hepatitis, unhygienic practices related to drinking water, unhygienic practices related food intake, unhygienic practices related sanitation, contact history of viral hepatitis, history of travel to endemic area, history of previous vaccination, and overcrowding in the household. The face and content validity of the questionnaire were assessed by experts and the relevant items under each domain were listed. These draft tools were sent to five public health experts for their opinion and they were asked to allocate a score out of 10, taking into consideration the relevance of each variable to be retained in the tool. The score given for each item by the experts were added and the quartile analysis was done. The variables in the upper three quartiles were retained in the tool. The retained item variables were worded and a scoring system was developed for each probable risk factor. The re-drafted tools were re-circulated among the five experts to seek their feedback on the wording and the scoring and based on their feedback, the final tool was developed (Additional file 1). It was prepared using simple language, avoiding technical terms as much as possible to ensure accurate and reliable responses from the participants. The questionnaire was then pre-tested to ensure its acceptability and understandability. Where required, corrections and improvements were made following the pre-testing process.

In the questionnaire, questions/statements regarding knowledge on viral hepatitis included the nature of the diseases, possible modes of transmission, possible risk factors for HAV, commonly affecting organs, common clinical features, and vaccination against HAV. Total score allocated for the entire section was 150 and the cutoff for poor knowledge was decided as the first quartile of the total score. In the case of children under 12 years of age parents’ or the guardians’ knowledge was assessed. The exposure to hygienic drinking water was assessed by 10 items on quality, accessibility and usage of water sources, safety of containers and purity and the total score was 45. ‘Unhygienic practices related to drinking water’ was defined according to an individual’s aggregated score within the first quartile of the total score. The exposure to hygienic sanitation was assessed by 13 items on quality, availability of water, availability of soap, hand washing and related practices, waste disposal, and waste segregation, and the total allocated marks were 45. ‘Unhygienic sanitary practices’ was defined according to an individual’s aggregated score within the first quartile of the total score. The exposure to hygienic food intake was assessed by 11 items on quality, safe storage, cooking practices, selection of food and maintaining temperature and the total score was 50. ‘Unhygienic food practices’ was defined according to an individual’s aggregated score within the first quartile of the total score. A score was allocated to each item of the above probable risk factors assessed and the total score depended on the type of items, type of response, and the number of possible responses. The cutoff for each probable risk factor was decided with the experts’ views. Contact history of patient with icterus during previous 1 month, travel to high risk countries during previous 1 month, stay out of district of the Gampaha district for more than 24 h during the past 1 month were assessed. Lack of vaccination against HAV was assessed by asking of receiving two doses of inactivated or activated vaccine.

The IAQ was pre-tested by administering it to 10 clinically suspected cases with HAV in another Hospital which was not included as a setting in the study. Two trained pre-intern medical graduates were selected as interviewers for the study. For the case group, data were collected from the eligible patients in the hospital. For the control group, the selected households were visited for data collection. Interviewers were trained on the administration of the questionnaire to ensure uniform and accurate process of data collection. They were instructed to read out the questionnaire in its exact form in order to ensure minimal effect of the interviewer on the outcome. The interviewers were blinded. In this way interviewer bias has been avoided during the administration of the questionnaire to a certain extent.

Blood samples were collected by nursing officers according to strict aseptic procedures within the first 2 days of admission to the relevant hospital. Once collected, correctly labelled samples were kept in the refrigerator until they were transported to the laboratory facility. Serum samples were stored in a specific storage in the laboratory facility under minus 20 Centigrade temperature until the testing was performed. Serological tests were performed in the Department of Microbiology, Faculty of Medicine, University of Colombo under direct supervision of a senior Microbiologist. Anti-HAV IgM test was done by AccuDiag ELISA kits for suspected HAV cases while both Anti-HAV IgM and IgG test were done for controls. These particular tests (Anti-HAV IgM and Anti-HAV IgG) had 99% specificity and 100% sensitivity.

Data analysis was performed by Statistical Package for Social Sciences version 16. Probable risk factors were determined by bivariate analysis and multiple logistic regression was performed in order to identify independent risk factors. The variables with probability value up to 0.20 in the bivariate analysis were included into the multivariate model [15]. The adjusted odds ratios (AOR), and 95% confidence intervals (CI) were calculated.

The Ethics Review Committee of the Medical Research Institute of Sri Lanka granted ethical clearance for the study and written informed consent was obtained. All procedures were complying with the ethical standards of the relevant national and institutional guidelines including the Helsinki Declaration of 1975, as revised in 2008 and the International Ethical Guidelines for Biomedical Research Involving Human Subjects in 2002.

## Results

Two hundred and fifty two individuals were recruited as cases and another 252 individuals as controls. Majority of the controls belonged to the age group of 11 to 20 (*n* = 106, 42%) while majority of the cases belonged to the age group of 21 to 30 (*n* = 69, 27.4%). Fifty six per cent (*n* = 141) among the cases were males while the proportion of males among the controls were 39.3% (*n* = 99). In both the groups, majority of the participants belonged to the Sinhalese and Buddhist religious group. Married and unmarried individuals were more or less equal among the cases while majority (*n* = 192, 76.19%) of the individuals were unmarried among the controls. There were 160 (63.3%) and 115 (45.6%) individuals who were educated up to Grade 10 among the cases and controls respectively.

There were statistically significant associations between the presence of HAV infection and being above 13 years of age, being a male, being Buddhist, being Sinhalese, being married, having a low family income, being previously or currently employed, living in an urban area, having a higher number of members in the family, sharing toilets with a higher number of members in the family, foreign trips and out visits from the district of Gampaha (Table 1).

**Table 1:** Association with HAV infection and selected socio demographic characteristics of the participants

Socio demographic characteristics	Cases (***N*** = 252) n (%)	Controls (*N* = 252) n (%)	OR (95%CI)	***p*** value
**Age**			2.02 (1.3–3.1)	
> 13 years	213 (84.5)	184 (73.0)		
≤ 13 years	39 (15.5)	68 (27.0)		0.002
**Sex**				
Male	141 (56.0)	99 (39.3)	1.9 (1.38–2.8)	
Female	111 (44.0)	153 (60.7)		0.001
**Marital Status**				
Unmarried	132 (52.4)	192 (76.2)	0.36 (0.24–0.53)	
Married	115 (45.6)	60 (23.8)		< 0.001
**Religion**				
Buddhism	250 (99.2)	219 (86.9)	18.8 (4.46–79.4)	
Catholic	2 (0.8)	33 (13.1)		< 0.001
**Ethnicity**				
Sinhala	252 (100.0)	246 (97.6)	2.02 (1.85–2.21)	
Tamil	0 (0.00)	6 (2.4)		0.01
**Family income**				
< 20,000 LKR	169 (85.8)	184 (73.0)	2.23 (1.37–3.63)	
≥ 20,000 LKR	28 (14.2)	68 (27.0)		0.001
**Highest level of education**				
Up to O/L	182 (76.5)	188 (74.6)	1.10 (0.73–1.67)	
Above O/L	56 (23.5)	64 (25.4)		0.63
**Occupation**				
Other	154 (61.1)	221 (87.7)	0.22 (0.14–0.36)	
Paid worker/Retired	98 (38.9)	31 (12.3)		0.001
Urban	101 (40.1)	36 (14.3)	4.01 (2.60–6.19)	
Rural	151 (59.9)	216 (85.7)		< 0.001
**Number of family members sharing the household**				
≥ 5 members	56 (22.2)	104 (41.3)	0.41 (0.28–0.60)	
< 5 members	196 (77.8)	148 (58.7)		< 0.001
**Family members sharing the toilet**				
≥ 5 members	137 (54.4)	179 (71.0)	0.49 (0.34–0.70)	
< 5 members	115 (45.6)	73 (29.0)		< 0.001
**Out visits from Gampaha**				
Yes	34 (13.5)	152 (60.3)	0.10 (0.06–0.16)	
No	218 (86.5)	100 (39.7)		< 0.001
**Travelled abroad**				
Yes	0 (0.0)	42 (16.7)	0.45 (0.41–0.50)	
No	252 (100)	210 (83.3)		< 0.001

*OR* odds ratio, *CI* confidence interval

A statistically significant association was observed between the poor knowledge regarding viral hepatitis, unhygienic practices related to drinking water, unhygienic sanitary practices, and unhygienic food practices with the presence of HAV infection by bivariate analysis (Table 2).

**Table 2:** Association of HAV infection with knowledge about viral hepatitis and practices

	Cases (***N*** = 252) n (%)	Controls (***N*** = 252) n (%)	OR (95%CI)	***p*** value
**Knowledge regarding viral hepatitis**				
Poor knowledge	92 (36.5)	35 (13.9)	3.56 (2.30–5.53)	
Good knowledge	160 (63.5)	217 (86.1)		< 0.001
**Practices related to drinking water**				
Unhygienic	88 (34.9)	54 (21.4)	1.97 (1.32–2.93)	
Hygienic	164 (65.1)	198 (78.6)		
**Sanitary practices**				
Unhygienic	97 (38.5)	53 (21.0)	2.35 (1.58–3.49)	
Hygienic	155 (61.5)	199 (79.0)		< 0.001
**Food practices**				
Unhygienic	110 (43.7)	40 (15.9)	4.10 (2.70–6.25)	
Hygienic	142 (56.3)	212 (84.1)		< 0.001
**History of Hepatitis vaccination**				
Not vaccinated	41 (16.3)	41 (16.3)	1.0 (0.62–1.60)	
Vaccinated	211 (83.7)	211 (8 3.7)		1.0

*OR* odds ratio, *CI* confidence interval

Living in an urban area, (AOR-5.9, 95%CI = 2.98–11.86), low income of the family (AOR 2.82, 95%CI = 1.30–6.13), poor knowledge regarding viral hepatitis (AOR 3.89, 95%CI = 1.97–8.05), unhygienic sanitary practices (AOR 2.73, 95%CI = 1.42–5.23), and unhygienic practices related to drinking water (AOR 2.67, 95%CI = 1.37–5.21) were found as independent risk factors for HAV infection (Table 3). Being unmarried, being unemployed, out visits from the district of Gampaha, more than five family members living in a household and a higher number of members sharing a toilet in the household were inversely associated with HAV infection (Table 3).

**Table 3:** Risk factors for HAV infection

Variable	Beta coefficient	AOR	95% CI for AOR		***p*** value
Urban population	1.78	5.94	2.98	11.86	< 0.001
Unmarried individuals	−1.35	0.26	0.14	0.46	< 0.001
Unemployed	−2.13	0.12	0.06	0.23	< 0.001
Income < 20,000 LKR	1.04	2.82	1.30	6.13	0.009
Poor knowledge regarding viral hepatitis	1.38	3.98	1.97	8.05	< 0.001
Unhygienic practices related to drinking water	0.98	2.67	1.37	5.21	0.004
Unhygienic sanitary practices	1.00	2.73	1.42	5.23	0.003
Out visits from district of Gampaha	−2.19	0.11	0.06	0.21	< 0.001
≥ 5 family members living in a household	−1.01	0.36	0.19	0.75	0.006
≥ 5 family members using the toilet	−0.66	0.52	0.27	1.00	0.05

*AOR* adjusted odds ratio, *CI* confidence interval

## Discussion

The study found that independent risk factors of HAV infection were poor knowledge regarding viral hepatitis, unhygienic practices related to drinking water, unhygienic sanitary practices, being in an urban area, and low income of the family.

One of the main preventive measures of viral hepatitis is avoidance of transmission for which knowledge regarding modes of transmission of the disease is vital. Assessment of knowledge regarding viral hepatitis is not commonly found in literature. We found that poor knowledge regarding viral hepatitis was an independent risk factor with the occurrence of HAV infection.

The present study depicted that the practices related to unhygienic drinking water were independent risk factors. Similar findings were observed a study conducted in Italy which had found that the surface water as a potential source for the HAV infection [16]. Three studies reported that usage of water from the public system was identified as a risk factor in Brazil [17], in France [18], and in Korea [19]. The common feature of all of these studies was that only one or two aspects were measured rather than focusing on the full spectrum of the risk profile under the practices related to unhygienic drinking water. In the current study, the risk of unhygienic drinking water was assessed as a composite variable including all aspects in relation to such practices.

Individuals who conformed to poor sanitary practices were at 2.72 times a risk than the individuals who follow proper sanity practices, according to the present study. One study conducted in Jordan reported that presence of public sewage system in the resident area was a protective factor for HAV infection [20]. In contrast availability of different types of toilets was not associated with HAV infection [20]. Another study conducted in Chile reported that neither the presence of a public sewage system nor the availability of a toilet were associated with HAV infection [21]. Availability of water and soap for toilet practices, hand washing and related practices, proper waste disposal and segregation were selected for the assessment of hygienic sanitation in the current study. Relevant questions were made in order to get the maximum information under the hygienic sanitation. Therefore, overall individual assessment regarding hygienic sanitation was possible in the current study rather than assessment of individual items. Further, this method was useful to perform unbiased assessment of individuals since overall situation regarding sanitation was evaluated.

People who live in urban sector had six times higher risk of HAV than people who live in rural sector. It is important to understand the reasons behind the risk of being in the urban sector in order to plan preventive strategies. Urban sectors are controlled by Urban Councils and Municipal Councils and facilities like pipe-borne water, waste management, and many other facilities for the improvement of living standard are provided. However, this risk may be due to unplanned urbanization and poor living conditions in some areas due to high population density where waste management is observed to be not up to the standard [22]. Rural population may be protected due to their safe practices. In contrast, living in a rural area was identified as a risk factor for HAV in three studies [23–25].

Low income was independently associated with the occurrence of HAV infection. Low income level links with socioeconomic status of individuals or family [21, 26]. Hence the occurrence of HAV infection would be associated with low level of socioeconomic status and it is important to understand its implications. According to our findings, the variable such as education, number of family members sharing the household or toilet were significantly associated with hepatitis A infection. However, those variables became non-significant in the multivariate regression model. Further two studies showed that high socioeconomic states had low risk of HAV infection [21, 25].

There were a few limitations in the present study. Data collection was only dependent on the information given by the participants. The authors attempted to clarify any doubt in data collection by adopting several measures for improving quality of data. Further comprehensive socioeconomic status assessment has not been done. And as for the strengths of our study, we recruited a representative sample to minimize selection bias. Further, we used standardized questionnaire and trained interviewers to collect data. Standard techniques were used for drawing blood, storage and laboratory analysis. All of these methods minimized information bias.

## Conclusions

Independent risk factors were living in an urban area, low income, poor knowledge regarding viral hepatitis, unhygienic practices related to drinking water and unhygienic sanitary practices. Apart from the poor knowledge regarding viral hepatitis being a risk factor, we confirmed the findings reported by other authors. Measures should be taken in order to increase knowledge on HAV among general public, work settings, schools and preventive/curative health care institutions. Practices related to safe water intake, personal hygiene, and proper sanitation should be improved through behavior-oriented programmes.

## Supplementary information

**Additional file 1.** Risk factors for viral hepatitis A Infection Questionnaire.

## Data Availability

The datasets used and/or analysed during the current study are available from the corresponding author on reasonable request.

## Abbreviations

AOR: adjusted odds ratios
CI: confidence intervals
HAV: Hepatitis A virus
IAQ: Interviewer administered questionnaire
