# Supplementary material for: Risk factors for viral hepatitis A infection in Gampaha District, Sri Lanka: an unmatched case control study
Source: BMC Public Health. 2020 Mar 18;20:357. doi: 10.1186/s12889-020-08490-2 (PMC7079469; doi:10.1186/s12889-020-08490-2)
Supplement: Supplementary file 1 — Additional file 1. Risk factors for viral hepatitis A Infection Questionnaire. [file 12889_2020_8490_MOESM1_ESM.docx]

**Additional file 1**

**Title of data: Risk factors for viral hepatitis A Infection** **Questionnaire**

**SECTION B – KNOWLEDGE**

**(Mark the appropriate response with√ )**

| **NO** | **QUESTIONS AND FILTERS** | | **CATEGORY** | | | |
| --- | --- | --- | --- | --- | --- | --- |
| **12** | Have you heard about a disease called Hepatitis A before**?**  **(If the answer is “No” then go to the Section C)** | | Yes | | |  |
|  |  |  | No | | |  |
|  |  |  |  | | |  |
| **13** | What kind of a disease is it? | | Communicable | | |  |
|  |  |  | Non-Communicable | | |  |
|  |  |  | Do not know | | |  |
| **14** | What is/are the possible ways of transmission?  (Multiple answers are allowed) | | By mouth | | |  |
|  |  |  | By air | | |  |
|  |  |  | By contaminated soil | | |  |
|  |  |  | By touch | | |  |
|  |  |  | By mosquito bite | | |  |
|  |  |  | Sexually | | |  |
|  |  |  | By contaminated blood | | |  |
| **15** | What is/are the involved organ in Hepatitis A?  (Multiple answers are allowed) | | Liver | | |  |
|  |  |  | Brain | | |  |
|  |  |  | Heart | | |  |
|  |  |  | Lungs | | |  |
|  |  |  | Kidney | | |  |
|  |  |  | Other | | |  |
| **16** | Is there a vaccine to prevent Hepatitis A? | | Yes | | |  |
|  |  |  | No | | |  |
|  |  |  | Do not know | | |  |
| **17** | If “Yes” is it available in Sri Lanka? | | Yes | | |  |
|  |  |  | No | | |  |
|  |  |  | Do not know | | |  |
| **18** | If “Yes” does it inject to everyone in Sri Lanka? | | Yes | | |  |
|  |  |  | No | | |  |
|  |  |  | Do not know | | |  |
| **19** | What are the possible risk factor/ factors?  (Multiple answers are allowed) | | Use unclean toilets | | |  |
|  |  |  | Consume contaminated water | | |  |
|  |  |  | Consume contaminated foods | | |  |
|  |  |  | Common use of plates with others | | |  |
|  |  |  | Talking to ill | | |  |
|  |  |  | Staying with infected | | |  |
| **20** | What are the possible symptoms of Hepatitis A? | | | |  |  |
|  | a. Yellowish discoloration of eyes | Yes | | No | Don’t know | |
|  | b. Abdominal pain | Yes | | No | Don’t know | |
|  | c. Nasal bleeding | Yes | | No | Don’t know | |
|  | d. Dark tea colour urine | Yes | | No | Don’t know | |
|  | e. Numbness over extremities | Yes | | No | Don’t know | |
|  | f. Fever | Yes | | No | Don’t know | |
|  | g. Pale stools | Yes | | No | Don’t know | |

**SECTION C – CLINICAL INFORMATION**

| **NO** | **QUESTIONS AND FILTERS** | **CATEGORY** | | | ***OFFICE USE ONLY** |
| --- | --- | --- | --- | --- | --- |
| **21** | Have you ever had hepatitis in your lifetime?  If answer is ‘No’ or ‘Don’t know’ Skip to the **section D** | Yes | No | Don’t Know |  |
| **22** | If yes do you know the diagnosis? | Yes | No | Don’t Know |  |
| **23** | If yes what is the diagnosis?  (Mark the appropriate answer) | Hepatitis A | |  |  |
|  |  | Hepatitis B | |  |  |
|  |  | Hepatitis C | |  |  |
|  |  | Hepatitis D | |  |  |
|  |  | Hepatitis E | |  |  |
|  |  | Not Certain | |  |  |

| **24** | Have you experienced following symptoms during your lifetime? | | |  |
| --- | --- | --- | --- | --- |
|  | 1. Yellowish discoloration of eyes | Yes | No |  |
|  | 1. Dark tea colour urine | Yes | No |  |
|  | 1. Pale stools | Yes | No |  |

**SECTION D – RISK FACTOR ASSESSMENT**

(Answers to this section should be given considering your practices of the last month unless specified in the question)

**D.1 HYGIENIC WATER DRINKING**

| **NO** | **QUESTIONS AND FILTERS** | **CATEGORY** | | | | ***OFFICE USE ONLY** |
| --- | --- | --- | --- | --- | --- | --- |
|  |  | **Never** | **Sometimes** | **Most of the time** | **Always** |  |
| **25** | Was the water you drank collected-from the water sources by a clean container? |  |  |  |  |  |
| **26** | Was the water you drank stored in a clean container? |  |  |  |  |  |
| **27** | Did you use a cleaned cup for drinking water? |  |  |  |  |  |
| **28** | Were the cups you used to drink water, shared with others? |  |  |  |  |  |
| **29** | Did the places that you got water for drinking have had a latrine within 50 feet? | | Yes | No | Don’t know |  |
| **30** | Were the water sources easily accessible to you? | | | Yes | No |  |
| **31** | Mention the distance in feets, between water source and your Residence? | | |  | |  |

| **32** | What was/ were the source/s of water you drinking? (Multiple answers are allowed) | Water board (Public supply) |  |  |
| --- | --- | --- | --- | --- |
|  |  | Bottled mineral water |  |  |
|  |  | Well |  |  |
|  |  | River/lake/reservoir |  |  |
|  |  | Other **(Specify) … … … … … … …** | |  |
| **33** | What type of water did you drink at home? (Multiple answers are allowed) | Boiled water |  |  |
|  |  | Filtered water |  |  |
|  |  | Both boiled and filtered |  |  |
|  |  | Without boiling and filtering |  |  |
|  |  | Other **(Specify) … … … … … … …** | |  |
| **34** | Have you consumed following water based food items during last one month?  (Multiple answers are allowed) | ‘Saruwath’ |  |  |
|  |  | ‘Ice packets’ |  |  |
|  |  | ‘Ice palam’ |  |  |
|  |  | ‘Kolakenda’ |  |  |
|  |  | ‘Milk packets’ |  |  |

**D.2 HYGIENIC FOOD INTAKE**

| **NO** | **QUESTIONS AND FILTERS** | **CATEGORY** | | | | ***OFFICE USE ONLY** |
| --- | --- | --- | --- | --- | --- | --- |
|  |  | **Never** | **Sometimes** | **Most of the time** | **Always** |  |
| **35** | Did you eat your main meals from reliable sources? |  |  |  |  |  |
| **36** | Did you get your snacks from reliable sources? |  |  |  |  |  |
| **37** | Did you read the food labels when buying food? |  |  |  |  |  |
| **38** | Did you wash your hands before handling food? |  |  |  |  |  |
| **39** | Did you wash your hands before eating food? |  |  |  |  |  |
| **40** | Were the utensils used in cooking the food you ate, cleaned regularly? |  |  |  |  |  |
| **41** | Was the kitchen which prepared the food you ate, free of insects and rodents? |  |  |  |  |  |
| **42** | Was raw food and cooked food stored separately in settings where you prepare food? |  |  |  |  |  |
| **43** | Are you satisfied on personal hygiene of the person who preparers the food at your home? | Yes | | |  |  |
|  |  | No | | |  |  |
|  |  | Not certain | | |  |  |
| **44** | Did you cover food items while storing at your home? | Yes always | | |  |  |
|  |  | Yes Frequently | | |  |  |
|  |  | Occasionally | | |  |  |
|  |  | Rarely | | |  |  |
|  |  | No | | |  |  |
|  |  | Do not know | | |  |  |
| **45** | Have you consumed following food items during last one month?  (Multiple answers are allowed) | Salads | | |  |  |
|  |  | Half boiled eggs | | |  |  |
|  |  | Raw vegetables | | |  |  |
|  |  | Semi cooked meat/fish | | |  |  |
|  |  | Semi cooked other foods | | |  |  |

**D.3 HYGIENIC SANITATION**

| **NO** | **QUESTIONS AND FILTERS** | **CATEGORY** | | | | ***OFFICE USE ONLY** |
| --- | --- | --- | --- | --- | --- | --- |
|  |  | **Never** | **Sometimes** | **Most of the time** | **Always** |  |
| **46** | Did you use a latrine for defecation? |  |  |  |  |  |
| **47** | Did you use a latrine for urination? |  |  |  |  |  |
| **48** | Did you use clean latrines? |  |  |  |  |  |
| **49** | Did the latrines you used, had water supply/papers cleaning for the toilet practices? |  |  |  |  |  |
| **50** | Were the latrines you used, cleaned regularly? |  |  |  |  |  |
| **No** | **QUESTIONS AND FILTERS** | **Never** | **Sometimes** | **Most of the time** | **Always** | ***OFFICE USE ONLY** |
| **51** | Did you use latrines that were not used by more than 5 people? |  |  |  |  |  |
| **52** | Was water available to clean hands after using the latrines? |  |  |  |  |  |
| **53** | Was soap available to clean hands after using the latrines? |  |  |  |  |  |
| **54** | Did you wash your hands with soap and water after using the toilet? |  |  |  |  |  |
| **55** | Was proper waste segregation done in settings you live, work or stay? |  |  |  |  |  |
| **56** | Was proper waste collection done in settings you live, work or stay? |  |  |  |  |  |
| **57** | Was waste removal done regularly in settings you live, work or stay? |  |  |  |  |  |
| **58** | What was the type of toilet at your home? |  | | Home | Work place |  |
|  |  | Water sealed commode type | |  |  |  |
|  |  | Water sealed squatting type | |  |  |  |
|  |  | Pit latrine | |  |  |  |
|  |  | No toilet facilities | |  |  |  |
|  |  | Other **(Specify)** … … … ………… … … … … | | | |  |

**D.4 CONTACT HISTORY**

| **NO** | **QUESTIONS AND FILTERS** | **CATEGORY** | | ***OFFICE USE ONLY** |
| --- | --- | --- | --- | --- |
| **59** | Have you exposed to a patient with yellowish discoloration of eyes during last month? | Yes |  |  |
|  |  | No |  |  |
|  |  | Don’t know |  |  |
| **60** | If yes, was it hepatitis? | Yes |  |  |
|  |  | No |  |  |
|  |  | Don’t know |  |  |
| **61** | If it’s hepatitis what was the diagnosis? | Hepatitis A |  |  |
|  |  | Other |  |  |
|  |  | Don’t know |  |  |

**D.5 TRAVEL HISTORY**

| **NO** | **QUESTIONS AND FILTERS** | **CATEGORY** | | ***OFFICE USE ONLY** |
| --- | --- | --- | --- | --- |
| **62** | Have you been out of district of Gampaha during last one month? | Yes |  |  |
|  |  | No |  |  |
| **63** | Have you visited a foreign country during the last one month? | Yes |  |  |
|  |  | No |  |  |
| **64** | If yes, what was / were the country / countries? |  | |  |

**D.6 VACCINATION HISTORY**

| **NO** | **QUESTIONS AND FILTERS** | **CATEGORY** | | ***OFFICE USE ONLY** |
| --- | --- | --- | --- | --- |
| **65** | Have you received the Hepatitis A vaccine in the past? | Yes |  |  |
|  |  | No |  |  |
|  |  | Don’t know |  |  |
| **66** | If ‘yes’ how many doses? |  | |  |

**D.7 CROWDING**

| **NO** | **QUESTIONS AND FILTERS** | **CATEGORY** | ***OFFICE USE ONLY** |
| --- | --- | --- | --- |
| **67** | How many family members stay in your residence? |  |  |
| **68** | How many members use the toilet that you were using during last one month? |  |  |
